# Early prediction of COVID‐19 severity using extracellular vesicle COPB2

**DOI:** 10.1002/jev2.12092

**Published:** 2021-06-02

**Authors:** Yu Fujita, Tokio Hoshina, Juntaro Matsuzaki, Yusuke Yoshioka, Tsukasa Kadota, Yusuke Hosaka, Shota Fujimoto, Hironori Kawamoto, Naoaki Watanabe, Kenji Sawaki, Yohei Sakamoto, Makiko Miyajima, Kwangyole Lee, Kazuhiko Nakaharai, Tetsuya Horino, Ryo Nakagawa, Jun Araya, Mitsuru Miyato, Masaki Yoshida, Kazuyoshi Kuwano, Takahiro Ochiya

**Affiliations:** ^1^ Department of Translational Research for Exosomes The Jikei University School of Medicine Tokyo Japan; ^2^ Division of Respiratory Diseases Department of Internal Medicine The Jikei University School of Medicine Tokyo Japan; ^3^ Department of Infectious Diseases and Infection Control The Jikei University School of Medicine Tokyo Japan; ^4^ Department of Molecular and Cellular Medicine Institute of Medical Science Tokyo Medical University Tokyo Japan; ^5^ Omiya City Clinic Saitama Japan

**Keywords:** biomarker, COVID‐19, extracellular RNA, extracellular vesicle, SARS‐CoV‐2

## Abstract

The clinical manifestations of COVID‐19 vary broadly, ranging from asymptomatic infection to acute respiratory failure and death. But the predictive biomarkers for characterizing the variability are still lacking. Since emerging evidence indicates that extracellular vesicles (EVs) and extracellular RNAs (exRNAs) are functionally involved in a number of pathological processes, we hypothesize that these extracellular components may be key determinants and/or predictors of COVID‐19 severity. To test our hypothesis, we collected serum samples from 31 patients with mild COVID‐19 symptoms at the time of their admission for discovery cohort. After symptomatic treatment without corticosteroids, 9 of the 31 patients developed severe/critical COVID‐19 symptoms. We analyzed EV protein and exRNA profiles to look for correlations between these profiles and COVID‐19 severity. Strikingly, we identified three distinct groups of markers (antiviral response‐related EV proteins, coagulation‐related markers, and liver damage‐related exRNAs) with the potential to serve as early predictive biomarkers for COVID‐19 severity. As the best predictive marker, EV COPB2 protein, a subunit of the Golgi coatomer complex, exhibited significantly higher abundance in patients remained mild than developed severe/critical COVID‐19 and healthy controls in discovery cohort (AUC 1.00 (95% CI: 1.00‐1.00)). The validation set included 40 COVID‐19 patients and 39 healthy controls, and showed exactly the same trend between the three groups with excellent predictive value (AUC 0.85 (95% CI: 0.73‐0.97)). These findings highlight the potential of EV COPB2 expression for patient stratification and for making early clinical decisions about strategies for COVID‐19 therapy.

## 1 INTRODUCTION

Severe acute respiratory syndrome coronavirus 2 (SARS‐CoV‐2) is responsible for a rapidly‐unfolding pandemic that is overwhelming health care systems worldwide (Dong et al., 2020). The majority of Coronavirus‐disease 2019 (COVID‐19) patients exhibit mild clinical symptoms, including fever, cough, and sputum. In some cases, more severe symptoms such as dyspnoea and/or hypoxemia occur after 1 week, with 50% of these severely‐affected patients quickly progressing to systemic, life‐threatening disorders, including acute respiratory distress syndrome (ARDS), septic shock, refractory metabolic acidosis, coagulation disorders, and multiple organ failure (Guan et al., 2020). These problems lead to both mild and severe respiratory manifestations, the latter being prominent in the elderly and in those with underlying medical conditions such as cardiovascular and chronic respiratory disease, diabetes, and cancer (Guan et al., 2020). The main clinical feature of severe COVID‐19 is the onset of ARDS (Huang et al., 2020). It has been reported that the rate of occurrence of ARDS in patients with severe COVID‐19 is 15.9%‐29% (Guan et al., 2020; Huang et al., 2020). The immune response that is vital for the control and resolution of SARS‐CoV‐2 infections can also lead to cellular damage in association with systemic deteriorations. Recent studies suggest that an exaggerated inflammatory response known as cytokine storm may play a key role in this process (Mangalmurti & Hunter, 2020). The variability of patient responses to SARS‐CoV‐2 infection makes it difficult to identify individuals who may be most at‐risk for severe responses to the disease and to create early strategies for treating the disease. Some studies have investigated their clinical and laboratory data to predict the development of severe events (Bi et al., 2020; Chen et al., 2020; Zhang et al., 2020). Furthermore, serum albumin, lactate dehydorogenase (LDH), C‐reactive protein (CRP), neutrophil counts, and D‐dimer were related with the development of severe COVID‐19 pneumonia (Al‐Samkari et al., 2020; Gong et al., 2020; Mo et al., 2020; Wang et al., 2020; Zhang et al., 2020). Although these clinical parameters provided important information to understand COVID‐19 outcomes, there is no reliable tool for effectively predicting disease severity or likely trajectories of illness progression (Chen et al., 2020).

Early identification of COVID‐19 patients who may experience severe disease deterioration is of great significance for improving clinical approaches that will result in reduced mortality. Accordingly, the purpose of our study has been to identify extracellular components that can serve as useful biomarkers for predicting disease progression in COVID‐19 patients. Toward this end we have focused on identifying the profiles of extracellular vesicle (EV) proteins and extracellular RNAs (exRNAs) in serum samples taken from COVID‐19 patients with mild symptoms at the time of their admission for medical treatment. 

Extracellular vesicles (EVs), including exosomes and microvesicles, contain numerous and diverse bio‐molecules such as RNAs and proteins within the vesicle's lipid bilayer (Yáñez‐Mó et al., 2015). This stable bilayer construction enables intact EVs to circulate through body fluids. Thus, EVs can transfer their cargo to target cells as a means of regulating target cell participation in both physiological and pathological processes, including host immune responses. EVs are characterized by a number of exosomal markers, including members of the tetraspanin family (CD9, CD63, CD81), components of the endosomal sorting complex required for transport (ESCRT) (TSG101, Alix), heat shock proteins (Hsp60, Hsp70, Hsp90), and RAB proteins (RAB27a/b) (Kowal et al., 2016). Accumulating evidence suggests that the size, membrane composition, and contents of EVs are highly heterogeneous, depending on the cellular source. Furthermore, EV cargoes can be dynamically altered by microenvironmental stimuli including viral infection. The EVs produced by both immune and non‐immune cells can be responsible for regulating the nature of the immune response as it relates to inflammation, autoimmunity, and infectious disease pathology (Robbins & Morelli, 2014). EVs derived from virus‐infected cells have been shown not only to modulate immune cell responses, but also to spread the viral infection via delivery of the viral genome and protein particles to healthy cells (Pegtel et al., 2010). Intriguingly, EVs have receptors for coronavirus entry such as CD9 (Earnest et al., 2017) and angiotensin‐converting enzyme 2 (ACE2) (Wang et al., 2020), suggesting that EVs may also have a role in modulating or mediating SARS‐CoV‐2 infection. For these reasons, evaluating the molecular profiles of circulating EVs in COVID‐19 patients may provide clues for understanding the host antiviral immune response and the mechanisms that determine disease trajectory and patient deterioration.

Studies of extracellular RNA (exRNA) have recently broadened into an important area of research with relevance to disease biomarker discovery and therapeutics (Das et al., 2019). Most exRNAs are protected from degradation in biofluids via incorporation into EVs or into complexes with lipids and proteins. The variety of exRNA species that have been identified include messenger RNAs (mRNAs) and non‐coding RNAs such as microRNAs (miRNAs), small nuclear RNAs, transfer RNAs, and long non‐coding RNAs (lncRNAs). During the early stages of viral infection, host non‐coding RNAs such as miRNAs are produced and released as a part of the antiviral response that is aimed either directly or indirectly at targeting viral transcription, translation, and replication (Tenoever, 2013). ExRNAs mediate a complex network of interactions between the virus and infected host cells, representing a pivotal role in modulating the host innate immune system (Girardi et al., 2018). These findings suggest that exRNAs may have the potential to serve as biomarkers for evaluating the antiviral responses mounted by COVID‐19 patients.

## 2 RESULTS

### 2.1 Baseline clinical characteristics of discovery cohort

For this retrospective, single‐centre study, 31 SARS‐CoV‐2 infected patients and 10 uninfected healthy donors were included in discovery cohort. At the time of patient admission for treatment, serum samples were taken from all 41 individuals as a source of material for identifying biomarkers (**Table S1**). The severity of COVID‐19 disease in each patient was graded using Clinical management of COVID‐19 interim guidance by WHO. At the time of admission, all COVID‐19 patients had a mild status. Based on the clinical course of disease progression after admission, we divided the 31 COVID‐19 patients into two groups: Group 1 included 22 patients who retained their mild status, and Group 2 included nine patients who progressed to severe/critical status (**Figure S1a**). All patients in Group 1 were subsequently discharged in good health from the hospital or transferred to the local medical facility for further observation. In Group 2, two patients (22.2%) died from COVID‐19 complications (pulmonary embolism (*n *= 1) or ARDS (*n *= 1)), and two patients (22.2%) remained hospitalized at the time of writing. All other patients in Group 2 were discharged in good health from the hospital following treatment (**Table S1**). Clinical parameters were comparable among the groups (**Table S2**). Healthy donors and COVID‐19 patients (Group 1 & 2) did not differ with respect to age, sex, body mass index (BMI), smoking index, levels of blood urea nitrogen (BUN), creatinine (Cr), and alanine aminotransferase (ALT), history of hypertension, diabetes mellitus, dyslipidaemia, and coronary heart disease (*P *> 0.05). Significant differences were observed in white blood cell (WBC) count and levels of CRP (*P* < 0.05). Comparison of COVID‐19 patients in Group 1 and Group 2 revealed no significant difference in sex, BMI, WBC count, BUN, Cr, creatine kinase (CK), D‐dimer, and fibrinogen, history of hypertension, diabetes mellitus, dyslipidaemia, and coronary heart disease (*P* > 0.05). However, significant differences were observed with respect to age, smoking index, and levels of CRP and ALT (*P* < 0.05). We selected these 4 parameters for Pearson's correlation analysis to identify correlations between the three groups in terms of age, smoking index, CRP, and ALT (**Figure S1b**). There were significant correlations in age, CRP, and ALT between the three groups (*P* trend < 0.05).

### 2.2 Predictive value of 4 clinical parameters for COVID‐19 severity

We performed receiver operating characteristic (ROC) analysis on combined Group1 and Group 2 to provide a robust test of sensitivity, specificity, and area under the curve (AUC) for our set of four predictive parameters (**Figure S1c**). The AUC values for the four factors (age, smoking index, CRP, and ALT) were 0.90 (95% CI: 0.79‐1.00), 0.69 (95% CI: 0.48‐0.89), 0.77 (95% CI: 0.60‐0.94), and 0.72 (95% CI: 0.51‐0.94), respectively. These findings suggest that at the time of admission to the hospital, age and CRP, having AUC values > 0.75, can provide the greatest discrimination between patients who will develop mild versus severe COVID‐19.

Based on the ROC analysis, optimal cutoffs for the 4 parameters were identified via use of the Youden index (**Table S5**). For further analysis, we used these cutoffs to separate COVID‐19 patients into low and high groups for comparing the incidence of severe COVID‐19 related events. Kaplan‐Meier curves were constructed for each of the four parameters to compare the high and low groups for time‐to‐onset of a severe event (beginning with the day of admission) (**Figure S1d**). The progression‐free times for age and CRP were significantly shorter in the high group than in the low group (*P *= 3.9 × 10^–6^; *P *= 0.014, respectively). These findings suggest that the parameters of patient age and CRP can be useful at the time of admission to predict the incidence of future severe COVID‐19 related events. Currently, age and/or CRP levels are consistently used in the clinic for risk stratification to predict the potential severity of COVID‐19 progression (Guan et al., 2020; Wang et al., 2020). Results with discovery cohort of 31 COVID‐19 patients are therefore representative of the overall status of patients infected with SARS‐CoV‐2.

### 2.3 LC‐MS analysis of proteome profiles from EVs in serum samples from COVID‐19 patients and uninfected controls

**FIGURE 1:**
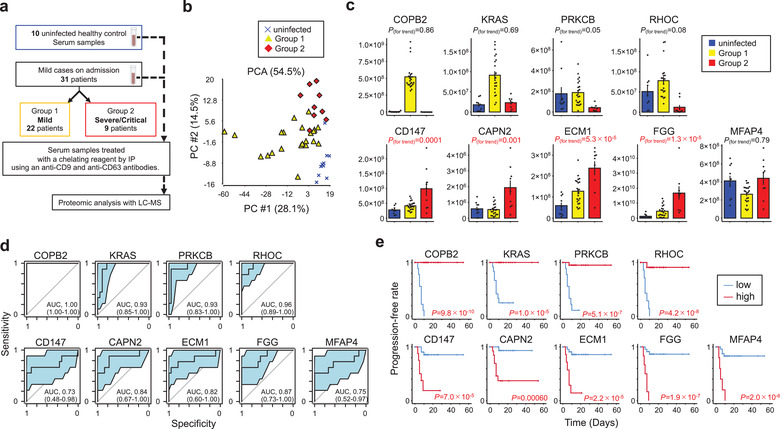
EV proteomes for early prediction of COVID‐19 severity in discovery cohort. [(**a**) Work flow for LC‐MS identification of proteomes from CD9^+^/CD63^+^ EVs obtained from serum samples of 31 mild COVID‐19 subjects (Group 1: *n *= 22, Group2: *n *= 9) and 10 uninfected healthy controls. (**b**) PCA map of 723 proteins from the three subject groups. (**c**) Correlations of COPB2, KRAS, PRKCM, RHOC, CD147, CAPN2, ECM1, FGG, and MFAP4 between the three subject groups. *P* values for trend by *Pearson's* correlation analysis. Error bars represent mean ± SEM. (**d**) AUC values (95% CI) for 9 EV proteins evaluated by ROC analysis. (**e**) Kaplan‐Meier curves for 9 EV proteins by Log‐rank test. Time represents the number of days from admission to time of onset for a severe COVID‐19 related event. Optimal cut‐off values were used to define high and low groups]

In clinical settings, analysis of EVs from liquid biopsies has gained attention as a potential means of identifying diagnostic and prognostic biomarkers for various diseases. However, this strategy has not yet been widely used due to a lack of standardized methods for isolating EVs from patients. To improve on this situation, we have employed an immunoprecipitation (IP) based method that targets surface marker proteins for rapid and specific isolation of EVs. The use of IP in the presence of a chelating reagent improves the yield and purity of CD9^+^ or CD63^+^ positive EVs from serum samples. We confirmed that scanning electron microscope (SEM) images of the immunocaptured vesicles showed 100–150 nm size vesicles are bound to the beads (**Figure S2a**). The purity of the EV marker positive vesicles was higher than ultracentrifugation method (**Figure S2b**). Furthermore, the lysed EVs have dramatically reduced amount of contaminant non‐EV marker proteins (ApoE and ApoA‐1) (**Figure S2c, d**). The resulting EV preparations are suitable for subsequent EV proteome analysis by LC‐MS (Figure 1a). From our total of 41 serum samples, LC‐MS analysis identified 1676 proteins, following exclusion of proteins that were not present in all samples. Of these 1676 proteins, a total 723 proteins were present at different levels between the three groups (*P *< 0.05; one‐way ANOVA). To compare the pattern of expression of these 723 EV proteins among the three patient cohorts, we used unsupervised multivariate statistics based on principal component analysis (PCA) mapping. PCA plots the first and second principal components (PC1 and PC2) using all the term frequency from the LC‐MS data, showing a certain degree of separated trends between the three groups (Figure 1b). This differentiation could be described by the first PC1, which accounted for 28.1% of the variance. The second PC2 accounted for 14.5% of the variance. This observation indicated that the EV proteome profiles of serum in COVID‐19 patients deviated considerably from those uninfected donors. Although a little overlap or dispersity was demonstrated, we found obvious EV proteomic differences between Group 1 and Group 2 in the PCA scores plot.

To further identify differences in EV proteins from Group 1 and Group 2 patients, a cross‐validation score (Urabe et al., 2019), which indicates the robustness of discrimination performance between them, was calculated on the basis of Fisher linear discriminant analysis for each of the selected proteins. From the candidate proteins, we have listed 91 proteins with cross‐validation scores > 0.75 (**Table S3**). Further, we show the abundance of the top 9 proteins with cross‐validation scores > 0.85 between the three patient groups (Figure 1c). Among these most‐abundant proteins, four proteins [COPI coat complex subunit beta 2 (COPB2), KRAS proto‐oncogene (KRAS), protein kinase C beta (PRKCB), and ras homolog family member C (RHOC)] are significantly more abundant in Group 1 than in Group 2 or in uninfected controls (*P* trend > 0.05). Furthermore, CD147, calpain 2 (CAPN2), extracellular matrix protein 1 (ECM1), fibrinogen gamma chain (FGG) were significantly more abundant in Group 2 than in uninfected controls or in Group 1 (*P* trend < 0.05). Only microfibril associated protein 4 (MFAP4) was less abundant in Group 1 than in uninfected controls or in Group 2 (*P* trend > 0.05).

### 2.4 Predictive value of 9 EV proteins for COVID‐19 severity

We performed ROC analysis for combined Group1 and Group 2 to provide a robust test of sensitivity, specificity, and AUC for our set of nine predictive markers (Figure 1d). The AUC values for the four markers with enhanced abundance in Group 1, COPB2, KRAS, PRKCM, and RHOC, were 1.00 (95% CI: 1.00‐1.00), 0.93 (95% CI: 0.85‐1.00), 0.93 (95% CI: 0.83‐1.00), and 0.96 (95% CI: 0.89‐1.00), respectively. AUC values for the other five markers, CD147, CAPN2, ECM1, FGG, and MFAP4, were 0.73 (95% CI: 0.48‐0.98), 0.84 (95% CI: 0.67‐1.00), 0.82 (95% CI: 0.60‐1.00), 0.87 (95% CI: 0.73‐1.00), and 0.75 (95% CI: 0.52‐0.97), respectively. Our analysis suggests that this set of markers, examined at the time of patient admission to the hospital, provides a significant degree of separation between patients that will develop mild versus severe COVID‐19.

Additional ROC curves were generated to identify optimal cutoff values for the 9 marker proteins according to the Youden index (**Table S5**). For further analysis, we used the cutoff values to separate COVID‐19 patients into low and high groups for comparing the onset of severe COVID‐19 related events. Kaplan‐Meier curves for the time‐to‐onset of a severe event were constructed for each of the 9 proteins (Figure 1 **e**). Progression‐free times for COPB2, KRAS, PRKCM, and RHOC, proteins with enhanced abundance in Group 1, were significantly shorter in the low group than in the high group (*P *= 9.8×10^–10^; *P *= 1.0×10^–5^; *P *= 5.1×10^–7^; *P *= 4.2×10^–8^, respectively). Conversely, the progression‐free times for CD147, CAPN2, ECM1, FGG, and MFAP4, proteins with enhanced abundance in Group 2, were significant shorter in the high group than in the low group (*P *= 7.0×10^–5^; *P *= 0.00060; *P *= 2.2×10^–5^; *P *= 1.9×10^–7^; *P *= 2.0×10^–6^, respectively). These results reinforce the concept that this set of markers can be valuable for predicting the likelihood of patients experiencing severe COVID‐19 related events.

### 2.5 NGS determination of exRNA profiles in serum samples from COVID‐19 patients and uninfected controls

**FIGURE 2:**
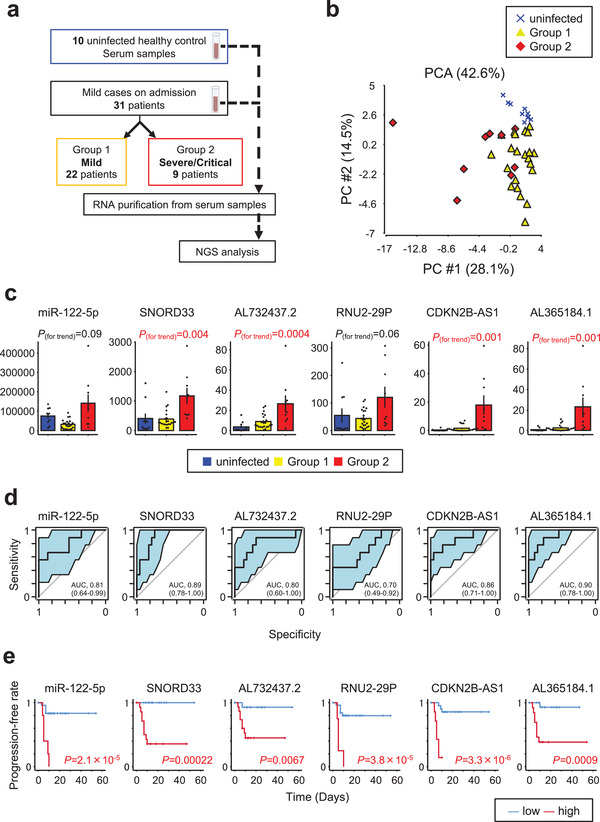
ExRNA profiles for early prediction of COVID‐19 severity in discovery cohort. [(**a**) Work flow for NGS determination of exRNA profiles from serum samples of 31 mild COVID‐19 patients (Group 1: *n *= 22, Group2: *n *= 9) and 10 uninfected healthy controls. (**b**) PCA map of 43 transcripts for the three subject groups. (**c**) Correlations of miR‐122‐5p, SNORD33, AL732437.2, RNU2‐29P, CDKN2B‐AS1, and AL365184.1 between the three subject groups. *P* values for trend by *Pearson's* correlation analysis. Error bars represent mean ± SEM. (**d**) AUC values (95% CI) for six transcripts evaluated by ROC analysis. (**e**) Kaplan‐Meier curves for six transcripts by Log‐rank test. Time represents the number of days from admission to time of onset for a severe COVID‐19 related event. Optimal cut‐off values were used to define high and low groups]

Circulating exRNAs have the potential to serve as biomarkers for a wide range of diseases. ExRNAs consist of diverse RNA subpopulations that are protected from degradation by incorporation into EVs or by association with lipids and/or proteins. ExRNA profiles in blood samples are dynamic and include mRNAs, miRNAs, piRNAs and lncRNAs (Murillo et al., 2019). Here we have used next‐generation sequencing (NGS) to analyze exRNAs present in patient serum samples (Figure 2 **a**). From our 41 serum samples, NGS analysis identified 408 transcripts, excluding transcripts with fewer than 50 reads in all samples. Of these exRNAs, 43 transcripts were differentially expressed between the three patient groups (*P *< 0.05; one‐way ANOVA). To identify patterns of expression for these 43 transcripts among the three groups, we performed unsupervised multivariate statistics based on PCA mapping. PCA plots from the NGS data revealed a trend toward separation between the three groups (Figure 2 **b**). This separation could be described by the first PC1, which accounted for 28.1% of the variance. The second PC2 accounted for 14.5% of the variance. These observations indicate that the serum exRNA profiles in COVID‐19 patients deviate considerably from those in uninfected donors. Moreover, despite some overlap or dispersity, the PCA plots allow us to detect obvious differences in exRNA profiles between Group 1 and Group 2.

For discrimination between Group 1 and Group 2, a cross‐validation score was calculated for each of the selected transcripts on the basis of Fisher linear discriminant analysis, much as described for our analysis of EV proteomes. From the candidate transcripts, we chose 14 transcripts with cross‐validation scores > 0.75 (**Table S4**). Figure 2 **c** compares the three patient groups for expression of the top 6 transcripts with cross‐validation scores > 0.80. These species include miR‐122‐5p, small nucleolar RNA C/D Box 33 (SNORD33), AL732437.2, RNA U2 small nuclear 29 Pseudogene (RNU2‐29P), CDKN2B antisense RNA1 (CDKN2B‐AS1), and AL365184.1 (this transcript has five different transcript IDs). Notably, the four transcripts SNORD33, AL732437.2, CDKN2B‐AS1, and AL365184.1 exhibit significantly higher levels of expression in Group 2 than in uninfected controls or Group 1 (*P* trend < 0.05).

### 2.6 Predictive value of 6 exRNAs for COVID‐19 severity

Next, we constructed ROC curves for Group1 and Group 2 to provide a robust test of sensitivity, specificity, and AUC values for our set of 6 predictive exRNA markers (Figure 2 **d**). AUC values for the transcripts miR‐122‐5p, SNORD33, AL732437.2, RNU2‐29P, CDKN2B‐AS1, and AL365184.1 were 0.81 (95% CI: 0.64‐0.99), 0.89 (95% CI: 0.78‐1.00), 0.80 (95% CI: 0.60‐1.00), 0.70 (95% CI: 0.49‐1.92), 0.86 (95% CI: 0.71‐1.00), and 0.90 (95% CI: 0.78‐1.00), respectively. This AUC analysis indicates that these exRNAs can provide a good basis for discriminating between mild and severe COVID‐19 cases at the time of patient admission.

Additional ROC curves were generated to identify optimal cutoff values for the 6 transcripts according to the Youden index (**Table S5**). Using these cutoff values, we separated COVID‐19 patients into low and high groups for determining the incidence of severe COVID‐19 related events. Kaplan‐Meier curves for time‐to‐onset of a severe event after admission were analyzed for each of the 6 transcripts (Figure 2 **e**). The progression‐free times for miR‐122‐5p, SNORD33, AL732437.2, RNU2‐29P, CDKN2B‐AS1, and AL365184.1 were all significantly faster in the high group than in the low group (*P *= 2.1×10^–5^; *P *= 0.00022; *P *= 0.0067; *P *= 3.8×10^–5^; *P *= 3.3×10^–6^; *P *= 0.0009, respectively). This finding suggests that these exRNA markers have value for predicting the incidence of severe COVID‐19 related events at the time of patient admission to the hospital.

### 2.7 The correlation between the selected markers for predicting disease severity values

Next, we used univariate Cox regression analysis to calculate the hazard ratio (HR) for each of the EV protein, exRNA, and clinical marker. Notably, the HR for COPB2 low was statistically incalculable using the optimal cut‐off value, suggesting that EV COPB2 has the best predictive value between the three sets of parameters. Precisely, age high (HR 28.1; 95% CI 3.4‐231.9; *P *= 0.0019), CRP high (HR 8.4; 95% CI 1–67.5; *P *= 0.045), PRKCB low (HR 32.1; 95% CI 3.9‐261.9; *P *= 0.0012), RHOC low (HR 23.6; 95% CI 4.7‐118; *P *= 0.00012), CD147 high (HR 10.7; 95% CI 2.5‐45.1; *P *= 0.0013), CAPN2 high (HR 15.5; 95% CI 1.9‐125.9; *P *= 0.010), ECM1 high (HR 11.6; 95% CI 2.8‐48.4; *P *= 0.00079), FGG high (HR 21.4; 95% CI 4.2‐110.4; *P *= 0.00025), MFAP4 high (HR 12.7; 95% CI 3.3‐48.6; *P *= 0.00022), miR‐122‐5p high (HR 10.5; 95% CI 2.7‐40.4; *P *= 0.00063), AL732437.2 high (HR 9.9; 95% CI 1.2‐79.9; *P *= 0.031), RNU2‐29P high (HR 10.4; 95% CI 2.6‐40.8; *P *= 0.00081), CDKN2B‐AS1 high (HR 14.4; 95% CI 3.4‐61.3; *P *= 0.00031), and AL365184.1 high (HR 14.2; 95% CI 1.8‐114.4; *P *= 0.013) were statistically significant (**Table S5**).

**FIGURE 3:**
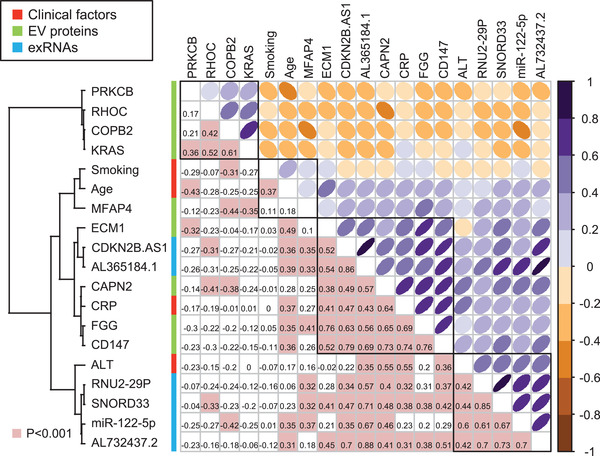
Upper triangular correlation plot of the associations between 4 clinical factors, 9 EV proteins, and 6 transcripts in discovery cohort. [Colours represent Pearson's correlation coefficients. Positive correlations are represented in purple, while negative correlations are represented in brown in the upper triangle. Colour intensity and ovalization of the circle are proportional to the correlation coefficients. The lower triangular correlation matrix displays actual correlation values, with pink highlights representing *P *< 0.05. Cluster 1 (PRKCB, RHOC, COPB2, and KRAS) contains a group of antiviral response‐related EV proteins. Clusters 2 (smoking, age, and MFPA4) and 3 (CM1, CDKN2B.AS1, AL365184.1, CAPN2, CRP, FGG, and CD147) contain groups of coagulation‐related markers. Cluster 4 (ALT, RNU2‐29P, SNORD33, miR‐122‐5p, and AL732437.2) contains a group of liver damage‐related exRNAs]

To investigate potential relationships between the selected markers, *Spearman's* correlation coefficients were calculated based on marker levels. A correlation plot was constructed to visualize the correlation coefficients of the 19 markers (Figure 3). This allowed identification of four hierarchical clusters of markers that share strong positive correlations within the groups to which they belong. Each marker appears to fit into one of the four well defined clusters (namely, cluster 1, 2, 3 and 4). Notably, we observe that cluster 1 (PRKCB, RHOC, COPB2 and KRAS) is negatively correlated with the other clusters. Clusters 2, 3, and 4 have substantially strong positive correlations with each other.

All 4 EV proteins in cluster 1 exhibit significantly higher abundance in Group 1 than in Group 2 COVID‐19 patients. This is consistent with the idea that cluster 1 might represent a group of antiviral response‐related EV proteins. Although the functions of RHOC or KRAS during SARS‐CoV‐2 infection remain unknown, there are some reports that implicate PRKCB or COPB2 in antiviral effects against SARS‐CoV‐2 (De Wilde et al., 2015; Li et al., 2020; Tsui et al., 2018).

Cluster 2 includes smoking, age, and MFPA4. MFAP4 is an extracellular matrix protein belonging to the fibrinogen‐related protein superfamily (Wulf‐Johansson et al., 2013). On the other hand, cluster 3 includes ECM1, CDKN2B.AS1, AL365184.1, CAPN2, CRP, FGG, and CD147. Based on previous evidences, we considered that ECM1, CDKN2B.AS1, CAPN2, and CD147 could be implicated in the abnormal coagulation status (Aguiar et al., 2020; Jang et al., 2010; Joghetaei et al., 2013; Steinhaeuser et al., 2020; Thomas et al., 2018). Reports of widespread thromboses and disseminated intravascular coagulation (DIC) in COVID‐19 patients have been rapidly increasing in number (Al‐Samkari et al., 2020). The levels of 1 exRNA (CDKN2B.AS1 from cluster 3) and 4 proteins relevant to extracellular matrix formation (MFPA4 from cluster 2 and ECM1, CAPN2, and CD147 from cluster 3) are correlated with the levels of FGG, which has crucial functions in coagulation (*P *< 0.05) (Figure 3). Taken together, the bulk of our data suggest that cluster 2 and 3 represent groups of coagulation‐related markers.

Cluster 4 components ALT, RNU2‐29P, SNORD33, miR‐122‐5p, and AL732437.2 may at least partly reflect phenomena related to liver damage (Bala et al., 2012; Michel et al., 2011; Rimer et al., 2018). Levels of ALT, a representative transaminase mainly associated with liver dysfunction, correlate with levels of the three exRNA species (*P *< 0.05). Recent data indicate that deranged expression of liver enzymes is one manifestation of COVID‐19 pathology, and that liver injury is more prevalent in severe cases than in mild cases of COVID‐19 (Hajifathalian et al., 2020; Zhang et al., 2020). Although the functions of the non‐coding RNAs RNU2‐29P and AL732437.2 are unknown, our data at least partially support the idea that exRNAs associated with liver damage can serve as biomarkers for predicting the severity of COVID‐19 in patients at the time of admission.

### 2.8 COPB2 as a vesicular protein localized inside EVs

**FIGURE 4:**
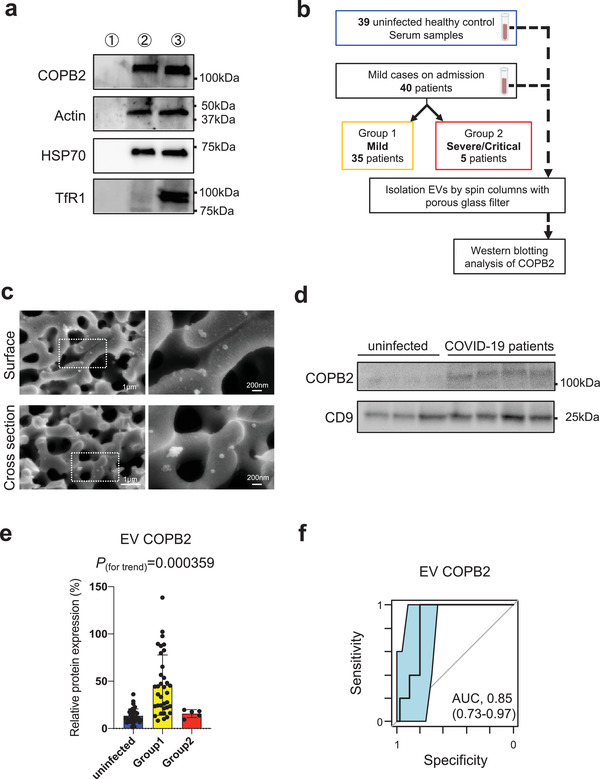
EV COPB2 for early prediction of COVID‐19 severity in validation cohort. [(**a**) The analysis for localization of COPB2 protein in EVs. The trypsin‐treated EVs were analyzed by Western blotting with antibodies against COPB2, Actin, Heat shock protein 70 (HSP70), and Transferrin receptor 1 (TfR1). Lane 1: PC9 EVs with membrane disrupting 0.2% Triton X‐100 for 10 min at room temperature before trypsin treatment, Lane 2: PC9 EVs with trypsin treatment, Lane 3: PC9 EVs without trypsin treatment. A 5 μg of EV proteins were loaded for detection of COPB2, Actin, and HSP70. A 3.5 μg of EV proteins were loaded for detection of TfR1. Actin and HSP70 are considered as a control of cytosolic protein, and TfR is considered as a control of integral membrane protein. (**b**) Work flow for Western blotting analysis of COPB2 in EVs isolated by spin columns with porous glass filter from the serum samples of 40 mild COVID‐19 subjects (Group 1: *n *= 35, Group2: *n *= 5) and 39 uninfected healthy controls. (**c**) SEM images of trapped EVs from serum samples in glass filter with nanoporous structure. (**d**) Representative image of Western blotting analysis of COPB2 and CD9 in EVs isolated by the columns from the serum samples of the groups. (**e**) Correlations of EV COPB2 protein levels between the three subject groups by Western blotting analysis. For the relative quantification of COPB2 protein expression between different experiments, we loaded 15 μg whole cell lysate from Jurkat cells as COPB2 normalized control (100%) for each experiment. *P* values for trend by *Pearson's* correlation analysis. Error bars represent mean ± SEM. (**f**) AUC values (95% CI) for EV COPB2 proteins evaluated by ROC analysis]

Among these markers in four clusters, finally we selected EV COPB2 protein, a subunit of the Golgi coatomer complex (Beck et al., 2009), with the best predictive value (AUC 1.00 (95% CI: 1.00‐1.00)) of predicting disease severity in discovery cohort. To clarify the existence of COPB2 as a vesicular protein, we performed trypsin‐digested proteomic approach on EVs. We manipulated a lung adenocarcinoma cell line PC9‐derived EVs which are positive for COPB2. EVs were isolated from PC9 cell culture supernatant by ultracentrifugation, and were treated with trypsin or 0.2% Triton X‐100 treatment. We used some markers, such as Actin and HSP70 considered as a control of cytosolic protein, and Transferrin receptor 1 (TfR1) considered as a control of integral membrane protein. Notably, COPB2, Actin, and HSP70 were not apparently affected by trypsin treatment but TfR1 was undetectable after trypsin treatment (Figure 4 **a**). The analysis indicated that COPB2 as a vesicular protein is localized insides EVs.

### 2.9 Predictive value of EV COPB2 proteins for COVID‐19 severity in validation cohort

Next, we investigated the predictive value of EV COPB2 in validation set consisting of 39 healthy controls and 40 patients with mild COVID‐19 symptoms at the time of their admission (**Table S6**). Based on the clinical course of disease progression after admission, we divided the 40 COVID‐19 patients into two groups in the same way as discovery set: Group 1 included 35 patients who retained their mild status, and Group 2 included five patients who progressed to severe/critical status (Figure 4 **b**). For the validation analysis, we use the spin column with porous glass filters which does not need any antibodies or chemical modifications, enabling rapid, simple, and efficient isolation of EVs with small centrifuge (the detail in supplementary information Methods). Indeed, SEM images showed the surface and cross‐sectional view of the porous structures after EV filtration. A number of EVs were adhered to the porous structure and the adhered 50–100 nm size vesicles exhibited intact morphology (Figure 4 **c**). To prove the purity of EVs by the spin column, we estimated the amount of contaminant non‐EV marker proteins, lipoproteins. We found that 1.8% ± 0.58% LDL/VLDL and 1.7% ± 0.94% HDL of serum samples remained in the extracted protein (**Figure S3a,b**). After removal of LDL/VLDL and HDL from serum samples, we found the COPB2 protein mainly in the isolated EV fraction, not LDL/VLDL (ApoB) or HDL (ApoA‐1), by spin column from the lipoprotein‐depleted serum (**Figure S3c**). These data suggested that the column would be suitable for detecting EV COPB2 in clinical setting.

Lysed EVs were analyzed by Western blotting using antibody specific to COPB2. We confirmed the consistent correlation of EV COPB2 protein levels between the groups in the Western blotting analysis with the column trapped EVs (Figure 4 **d**). In validation set, we confirmed that EV COPB2 protein were significantly more abundant in Group 1 than in Group 2 or in uninfected controls (Figure 4 **e**). Furthermore, we performed ROC analysis for combined Group1 and Group 2, and the AUC values was 0.85 (95% CI: 0.73‐0.97); sensitivity, 1.00 (95% CI: 0.57‐1.00); and specificity, 0.80 (95% CI: 0.64‐0.90) (Figure 4 **f**). Taken together, these findings suggested that the expression of EV COPB2 could serve as the predictive biomarkers for COVID‐19 disease severity.

## 3 DISCUSSION

Our results indicated the potential of EV COPB2 as a predictive biomarker for COVID‐19 disease severity in both the discovery and validation cohorts and may show utility for improved patient stratification and making early clinical decisions about strategies for COVID‐19 therapy.

COPB2 is a subunit of the Golgi coatomer complex I (COPI) that is necessary for retrograde trafficking from the Golgi to the endoplasmic reticulum (ER) (Beck et al., 2009; Szul & Sztul, 2011). Many viruses, including RNA viruses, DNA viruses, and retroviruses, hijack or adapt COPI‐related proteins for their own benefit. In view of the functions of COPI in infected cells, the subunits are specifically required for replication by a wide variety of viruses. In this context, EV COPB2 expression in viral infection might reflect the host factors that stimulate or restrict viral replication. In fact, COPB2 is required for replication of SARS‐CoV‐1, which is closely genetically related (79% identity) to SARS‐CoV‐2 (Lu et al., 2020). Depletion of COPB2 has a strong antiviral effect, based on reduced SARS‐CoV‐1 protein expression. Further studies are needed to elucidate whether EV COPB2 is a specific predictive biomarker for SARS‐CoV‐2 virus infection. Although the mechanistic insights of COPB2 packaged inside EVs still remain unclear, our data indicated that secretion of the EVs reflects the patient antiviral responses against SARS‐CoV‐2 infection.

Our research has implications that go beyond the simple investigation of biomarkers, since the results also provide important clues regarding the pathogenesis of COVID‐19 and the development of therapies for the disease. Indeed, not only COPB2 but also PRKCB and CD147 may be involved in SARS‐CoV‐2 infection or replication. PRKCB can regulate metabolic and mitochondrial aspects of reprogramming responsible for B cell fate (Tsui et al., 2018). A recent bioinformatics‐based report has revealed that PRKCB is one of the target genes activated by vitamin A that may have antiviral potential against SARS‐CoV‐2 (Li et al., 2020). Remarkably, CD147, known as extracellular matrix metalloproteinase inducer (EMMPRIN), is a transmembrane glycoprotein considered to be a binding partner for the SARS‐CoV‐2 spike protein, with obvious functional implications for viral infection (Aguiar et al., 2020). In terms of interaction with serious complications in COVID‐19 patients, 6 markers (MFAP4, ECM1, CDKN2B.AS1, CAPN2, FGG, and CD147) and 2 exRNAs (SNORD33 and miR‐122‐5p) are involved in thrombosis or liver injury. These findings indicate that the profiles of EV proteins and exRNAs in patient sera clearly reflect specific host reactions to SARS‐CoV‐2 infection and progression of the disease. Although the pathological significance of some markers is unknown, understanding the profiles of functional extracellular components in patient sera may help clarify various aspects of COVID‐19 pathogenesis. Therefore, this study will form the basis for more detailed follow‐up studies that should contribute to a better understanding of coronavirus‐host interactions and SARS‐CoV‐2 infection.

With regard to therapy, EV biomarkers may provide information concerning potential therapeutic targets for mitigating the effects of SARS‐CoV‐2 infection. For instance, our finding that 4 EV proteins in cluster 1 (COPB2, PRKCB, RHOC, and KRAS) are significantly more abundant in Group 1 than in Group 2 suggests that these proteins could have antiviral effects. Supplementation with these EVs might protect patients against COVID‐19 progression and the associated complications. In addition, a recent study showed that antibody against the spike protein receptor CD147 could block infection by SARS‐CoV‐2 (Aguiar et al., 2020). Similarly, neutralization or manipulation of other markers with higher abundance in Group 2 than in Group 1 might also have antiviral effects or provide protection against severe consequences of COVID‐19 progression.

As a single centre study with a small sample size, our work has several limitations, and caution should be exercised in utilizing the predictive value of EV COPB2. An expanded random sample across all genders and ages should be more representative of the general population, and larger clinical studies are required to validate the potential of these biomarkers for predicting the severity of COVID‐19 progression. A multicentre prospective study is ongoing to confirm the real value of EV COPB2.

In summary, our comprehensive evaluation identifies three distinct groups of components (antiviral response‐related EV proteins, coagulation‐related markers, and liver damage‐related exRNAs) capable of serving as early predictive biomarkers for the severity of COVID‐19 progression. Among these markers, COPB2 inside EVs has the best predictive value for severe deterioration of COVID‐19 patients both in discovery cohort and validation cohort. COVID‐19 patients with high levels of EV COPB2 at the time of admission might be able to overcome the disease without experiencing severe events. This study for the first time provides extracellular component profiles as potential resources for early discrimination between COVID‐19 patients that may be resistant to disease progression and patients that are likely to experience severe COVID‐19 related deterioration. Our results also suggest that, in addition to their predictive value, functional extracellular components can also be potential contributors and mitigators of pathogenesis during COVID‐19 deterioration.

## 4 METHODS

### 4.1 Study approval

This retrospective study involving collection of COVID‐19 serum samples was approved by the Institutional Review Board at The Jikei University School of Medicine (Number: 32–055(10130)). For discovery set, the protocol did not require informed consent, and patients were given the choice of opting out of the study. For validation set, written informed consent was provided by all COVID‐19 patients before sample acquisition. For collection of healthy control serum samples, this study was approved by the Institutional Review Board at The Institute of Medical Science, The University of Tokyo (Number: 28‐19‐0907). Written informed consent was provided by all healthy donors before sample acquisition, in accordance with Declaration of Helsinki principles.

## CONFLICTS OF INTEREST

Yu Fujita is a chief science advisor, Mitsuru Miyato is a chief executive officer, and Takahiro Ochiya is a technical executive officer of International Space Medical Co., Ltd. The other authors have declared that no conflict of interest exists.

## AUTHOR CONTRIBUTIONS

Yu Fujita and Takahiro Ochiya conceived the idea and coordinated the project. Yu Fujita, Tokio Hoshina, Juntaro Matsuzaki, Yusuke Yoshioka, Tsukasa Kadota, Yusuke Hosaka, Shota Fujimoto, Hironori Kawamoto, and Naoaki Watanabe performed statistical data analysis and *in vitro* experiments. Tokio Hoshina, Kenji Sawaki, Yohei Sakamoto, Makiko Miyajima, Kwangyole Lee, Kazuhiko Nakaharai, Tetsuya Horino, Ryo Nakagawa, and Masaki Yoshida collected serum samples. Mitsuru Miyato, Jun Araya, and Kazuyoshi Kuwano provided helpful discussion. The manuscript was finalized by Yu Fujita with the assistance of all authors.

## Supporting information

Supporting information.

Supporting information.
