# Supplementary material for: Early prediction of COVID‐19 severity using extracellular vesicle COPB2
Source: J Extracell Vesicles. 2021 Jun 2;10(8):e12092. doi: 10.1002/jev2.12092 (PMC8172627; doi:10.1002/jev2.12092)
Supplement: Supplementary file 1 — Supporting information. [file JEV2-10-e12092-s002.docx]

**Supplementary Information**

**Methods**

**Study subjects**

For discovery set, we enrolled 42 patients positive for SARS-CoV-2 who were admitted to the Jikei University Hospital between March and May 2020 (approved by the Institutional Review Board at the Jikei University School of Medicine (Number: 32-055(10130)). COVID-19 patients were recruited on the basis of positive PCR tests for SARS-CoV-2 RNA obtained by nasopharyngeal swabs. The eventual degree of COVID-19 severity was categorized as mild or severe/critical on the basis of Clinical management of COVID-19 interim guidance by WHO. 11 Patients with non-mild COVID-19 at admission were excluded. At the time of admission, all COVID-19 patients had a mild status. Based on the clinical disease course after admission, we divided 31 patients in two groups (namely, Group 1: mild cases, and Group 2: severe/critical cases). All patients received symptomatic treatment without corticosteroids according to Clinical management of COVID-19 interim guidance by WHO. 10 healthy age-matched donors were recruited at Omiya City Clinic, Saitama for routine medical examination between March and April 2019 (approved by the Institutional Review Board at The Institute of Medical Science, The University of Tokyo (Number: 28-19-0907).

For validation set, we enrolled 51 patients positive for SARS-CoV-2 who were admitted to the Jikei University Hospital between July and October 2020. 11 Patients with non-mild COVID-19 at admission were excluded. At the time of admission, all 40 COVID-19 patients had a mild status. We divided the mild patients in two groups (Group1 or Group2). All Group2 patients in validation set received dexamethasone and remdesivir which were approved in Japan. 39 healthy age-matched donors were recruited at Omiya City Clinic, Saitama for routine medical examination between March and April 2019.

The medical records of COVID-19 patients and healthy donors were analyzed retrospectively. The serum samples taken at admission from COVID-19 patients and from healthy donors were separated by centrifugation at 3000 rpm for 10 min at 4°C. The supernatant was collected into a new tube and the serum sample was stored at -80°C until use.

**Isolation of EVs for discovery set analysis**

Anti-CD9 antibody and anti-CD63 antibody (H.U. Group Research Institute, Tokyo, Japan) coupled to Dynabeads M-280 Tosylactivated (Thermo Fisher Scientific Inc, Waltham, MA, USA) were added to aliquots (500 μL) of the serum samples that had been treated with chelate-based PEVIA^®^ reagent (H.U. Group Research Institute), followed by incubation on a rotator at 4°C for 18 h according to the manufacturing protocol. The beads were washed three times with PBS and stored at 4°C until further analysis.

**Preparation of peptides for LC-MS**

EVs were processed using S-Trap micro spin columns (AMR Inc, Tokyo, Japan) according to the manufacturer’s instruction with minor modifications. In brief, captured EVs were suspended in 50 μL of 5% SDS (FUJIFILM Wako Pure Chemical Corporation, Osaka, Japan) in 50mM TEAB buffer (Honeywell Inc, Charlotte, NC, USA), pH7.5. After removing beads, the amount of protein from EVs were determined by Micro BCA™ Protein Assay Kit (Thermo Fisher Scientific Inc). 13.8 ng Pierce™ Digestion Indicator for Mass Spectrometry (Thermo Fisher Scientific Inc) was added to lysed samples for quality control of digestion efficiency. The samples were then reduced and alkylated with dithiothreitol (FUJIFILM Wako Pure Chemical Corporation) and iodoacetamide (Nacalai tesque Inc, Kyoto, Japan), respectively. 12% aqueous phosphoric acid (FUJIFILM Wako Pure Chemical Corporation) was added to a final concentration of 1.2% followed by six times the volume of S-Trap protein binding buffer. The sample mixtures were added to S-Trap columns that were prewashed and preconditioned with 0.2 % formic acid in 50 % acetonitrile and S-Trap buffer, respectively.　The S-Trap columns were washed with 150 μL S-Trap buffer. Centrifugation and removal of the flow through were then repeated 6 times. Washed columns were incubated with 20 μL of digestion buffer containing 0.75ug Trypsin/Lys-C Mix, Mass Spec Grade (Promega Corporation, Madison, WI, USA) for 2hr at 47°C. After digestion, peptides were eluted from the S-Trap column, lyophilized with miVac system (Genevac Ltd, Ipswich, United Kingdom), and stored at -80 °C until use.

**Proteomic analysis with LC-MS for discovery set analysis**

Peptides obtained from EV proteins were reconstituted in 10 μl of water containing 0.1% formic acid (FA) (Fisher Chemical, Thermo Fisher Scientific Inc). Quantification of peptides was accomplished using Pierce™ Quantitative Fluorometric Peptide Assay (Thermo Fisher Scientific Inc). Proteomic analysis of the peptides was carried out using Q Exactive (Thermo Fisher Scientific Inc.) equipped with UltiMate 3000 Nano LC Systems (Thermo Fisher Scientific Inc.). Peptide samples (1 μg) were injected onto Acclaim PepMap 1000 trap columns (75 μm × 2 cm, nanoViper C18 3 μm, 100Å, Thermo Fisher Scientific Inc) which were heated to 40 °C in a chamber which was connected to a C18 reverse-phase Aurora UHPLC Emitter Column with nano Zero & Captive Spray Insert (75 μm × 25 cm, Ion Opticks Pty Ltd) using Dreamspray interface (AMR Inc). The nano pump flow rate was set to 250 nL/min with a 302 min gradient, in which the mobile phases were A (0.1% FA in water, Fisher Chemical, Thermo Fisher Scientific Inc.) and B (0.1% FA in acetonitrile, Fisher Chemical, Thermo Fisher Scientific Inc.). The chromatography gradient was designed to provide a linear increase from 0-8 min at 2% B, 8-272 min from 2% B to 35% B, 272-282 min from 35% B to 70% B, 282-283 min from 70% B to 95% B, wash, 8 min and 10 min equilibrium. The data-dependent acquisition was performed in positive ion mode. Mass spectrometry parameters and those of the Proteome Discoverer 2.2.0.388 software (Thermo Fisher Scientific Inc) were described in a previous report ^1^.

**Immunoprecipitation of EVs and ELISA**

Human serum (Tennessee Blood Services) was used for apolipoprotein ELISA　assay. The antibodies conjugated to beads were added to the serum sample solution treated with PEVIA reagent (H.U. Group Research Institute) and incubated on a rotator for 18 h at 4°C according to the manufacturing protocol. Then, 80 µL of BRUB (Britton & Robinson Universal Buffer) pH 2.0 was added to the immunoprecipitated sample, incubated for 5 min at room temperature, and neutralized by adding 20 µL of 1M Tris-HCl Buffer. These samples were used for apolipoprotein ELISA. ELISA assays for ApoE (Proteintech, Rosemont, IL, USA) and ApoA-1 (Proteintech) were performed according to the manufacturer's instructions.

**Analysis of serum exRNA profiles for discovery set analysis**

Total RNA was extracted from aliquots (200 μL) of the serum samples using QIAzol and the miRNeasy Mini Kit (Qiagen, Hilden, Germany) according to the manufacturer's protocol. The library was prepared using the QIAseq miRNA Library Kit (Qiagen). Library preparations were subjected to quality control using either a Bioanalyzer 2100 or TapeStation 4200 system (Agilent Technologies, Santa Clara, CA, USA). The library pools were quantified using the Library Quantification Kit (Takara, Shiga, Japan) and sequenced on the NovaSeq 6000 sequencing platform (Illumina Inc, San Diego, CA, USA). Reads were pre-processed and annotated against miRBase v22.1 and Ensembl non-coding RNA database release 100 by using CLC Genomics Workbench v20.0.1. Raw and normalized microarray data is available in the Gene Expression Omnibus database (GSE158877).

**Isolation EVs by spin column with porous glass filter for validation set analysis**

A spin column with porous glass filter has been developed by Nagoya university and AGC Inc (Tokyo, Japan), resulting in highly efficient and easy to use EV isolation ^2,3^. Briefly, the filters were fabricated through spinodal decomposition. The pore size of the glass filter was accurately controlled by changing heating temperature and time. The glass filter having nanoporous structure was embedded into a spin column, and a 500 μL serum sample was filtered by spinning at 6,000×g. Trapped EVs were lysed by a lysis buffer and EV proteins were released inside the glass filter without EV recovery. For detecting lipoprotein contamination in the extracted EV fraction, we used HDL and LDL/VLDL Cholesterol Assay Kit (Cell Biolabs Inc, San Diego, CA, USA) according to the manufacturer’s instruction.

**EV visualization using a scanning electron microscope (SEM)**

Regarding immunocaptured EVs, the antibodies conjugated to beads were added to an aliquot (500 µL) of human serum (Tennessee Blood Services) treated with PEVIA reagent (H.U. Group Research Institute), incubated on a rotator for 18 h at 4°C according to the manufacturing protocol, and washed three times with PBS. The samples were observed using a field emission SEM system (JSM-7500F, JEOL Ltd, Tokyo, Japan). Regarding EVs captured in the spin column, the EVs were prepared by fixation with 2% glutaraldehyde buffered in PBS for 90 min and post-fixed in 1.5% osmium tetroxide for 60 min. Samples were next dehydrated in graded ethanol concentrations and then in graded t-Butyl alcohol. They were freeze-dried and coated with platinum using plasma chemical vapor deposition (CVD), and then observed using a field emission SEM system (JSM-6301F, JEOL Ltd).

**Isolation of EVs for *in vitro* analysis**

Human lung cancer cell lines PC9 (lung adenocarcinoma) were purchased from ATCC. PC9 cells were maintained in RPMI 1640 medium with 10% heat-inactivated fetal bovine serum and an antibiotic-antimycotic at 37 °C in 5% CO_2_. PC9 cells were washed with PBS and the culture medium was replaced with advanced RPMI1640 medium (Thermo Fisher Scientific Inc) containing an antibiotic-antimycotic and 2 mM L-glutamine. After incubation for 48 h, the conditioned medium (CM) was collected and centrifuged at 2,000 g for 10 min at 4 °C. To thoroughly remove cellular debris, the supernatant was filtered through a 0.22 μm pore-sized membrane filter (Stericup Quick Release Durapore, Merck Millipore, Burlington, MA, USA). To prepare EVs, the CM was ultracentrifuged at 210,000 × g (35,000 rpm) using a SW41Ti rotor (Beckman Coulter, Inc, Brea, CA, USA) for 70 min at 4 °C. The pellets were washed with 11 mL of PBS by ultracentrifugation at 210,000 × g (35,000 rpm) using the SW41Ti rotor for 70 min at 4 °C and resuspended in PBS.

**Western blotting**

EVs isolated from serum were suspended in 100 μL M-PER regent (Thermo Fisher Scientific Inc) with protease inhibitor cocktail (Sigma-Aldrich, Missouri, USA, #05892970001). For each experiment, equal amounts of EV proteins were resolved by 4-20% gradient SDS-PAGE gels. Subsequently, the gels were transferred to a polyvinylidene difluoride (PVDF) membrane (Merck Millipore, Burlington, MA, USA), and incubation with specific primary antibodies (rabbit anti-COPB2 antibody (BETHYL, Montgomery, TX, USA, #A304-522A), mouse anti-ApoA-1 antibody (Santa Cruz Biotechnology, Dallas, TX, USA, #sc-376818), mouse anti-ApoB antibody (Santa Cruz Biotechnology, #sc-13538), mouse anti-CD9 antibody (Santa Cruz Biotechnology, #sc-59140), mouse anti-actin antibody (Merck Millipore, #MAB1501, Clone C4), mouse anti-HSP70 antibody (BD Biosciences, California, USA, #610607, Clone 7/Hsp70), and mouse anti-TfR antibody (Thermo Fisher Scientific Inc, #136800, Clone H68.4) was performed for 1 h at 37 °C. After washing several times with PBST, the membrane was incubated with anti-rabbit IgG, HRP-linked secondary antibody (Cell Signaling Technology, Danvers, MA, USA, #7074) or anti-mouse IgG, HRP-linked secondary antibody (Cell Signaling Technology, #7076) followed by chemiluminescence detection (Thermo Fisher Scientific Inc, #34080) with the ChemiDocTM Touch Imaging System (BIO-RAD, California, USA). For the relative quantification of COPB2 protein expression between different experiments, we loaded an equal cell lysate from Jurkat cells (ATCC, Manassas, VA, USA) as COPB2 normalized control for each experiment. Regarding the experiment with lipoprotein-depleted serum in Figure S3c, we use LDL/VLDL and HDL Purification Kit (Cell Biolabs Inc) according to the manufacturer’s instruction.

**Trypsin digestion to EV proteins**

Approximately 20 μg of isolated EVs from CM of PC9 cells were incubated with 2 μg trypsin (Promega, Wisconsin, USA) for 3 h at 37°C and the reaction was stopped by addition of protease inhibitor cocktail (Sigma-Aldrich). After this treatment, the samples were denatured in 4X SDS sample buffer (Merck Millipore) for 10 min at 95°C and analyzed by Western blotting. Control EVs underwent exactly same procedure as trypsin-treated EVs in the absence of the trypsin. For disruption of EV membrane, EVs were incubated with 0.2% Triton X-100 (Sigma-Aldrich) for 10 min at room temperature before trypsin treatment.

**Statistical analysis**

Fisher’s exact test for categorical variables and unpaired Student’s *t*-test for continuous variables were used to compare clinical data between two groups. To identify biomarker candidates among EV proteins and exRNAs, we initially used one-way analysis of variance (ANOVA) to select candidates present at different levels in the three subject groups (uninfected, COVID-19 Group 1, and Group 2) with *P*< 0.05. Principal component analysis (PCA) was performed with the selected candidates using Partek Genomics Suite 7.0 (Partek, St. Louis, MO, USA). Second, candidates with better discrimination between Group 1 and 2 were selected based on linear discriminant analysis with leave-one-out cross-validation, and subsequent ROC analysis was performed using R version 3.6.3 (R Foundation for Statistical Computing, http:// www.R-project.org), compute.es package version 0.2-2, hash package version 2.2.6.1, MASS package version 7.3-51.5, mutoss package version 0.1-12, and pROC package version 1.16.2. Optimal cut-off values for each candidate were set based on the maximum point of the sum of sensitivity and specificity (Youden index). Predictive sensitivity, specificity, and accuracy were calculated with the corresponding cut-off value for each candidate. Kaplan–Meier analysis with log-rank test and Cox regression analysis were performed using IBM SPSS Statistics 25 (IBM Japan, Tokyo, Japan). The correlation plot was generated using R version 3.6.3 and corrplot package version 0.84., and the unsupervised hierarchical clustering analysis was performed using Partek Genomics Suite 7.0. The limit of statistical significance for all analyses was defined as a two-sided P value of 0.05.

**References**

1. bioRxiv, Kurimoto, A., *et al*. Enhanced recovery of CD9-positive extracellular vesicles from human specimens by chelating reagent. Preprint at <https://doi.org/10.1101/2020.06.17.155861> (2020).

2. Aoki, K., *et al*.  Exosome isolation toward cancer diagnosis using glass filter with nanoporous structure. *22^nd^ International Conference on Miniaturized Systems for Chemistry and Life Sciences.*2018 Nov 11-15, Kaohsiung, Taiwan, 1409-10.

3. Hatta, T., *et al*. P2.01-91 Exosomal analysis of ALK rearrangements by spin column with porous Glass filter. *J Thorac Oncol* 14, S676-S676 (2019).

**Table S1**. Baseline characteristics of healthy donors and COVID-19 patients in discovery study.

**Table S2**. Differences in clinical characteristics among healthy donors and COVID-19 patients in discovery study.

**Table S3**. EV proteins for discrimination between mild and severe COVID-19 patients in discovery study.

**Table S4**. ExRNAs for discrimination between mild and severe COVID-19 patients in discovery study.

**Table S5**. Univariate Cox regression analysis of the selected factors and biomarkers for

predicting COVID-19 severity in discovery study.

**Table S6**. Baseline characteristics of healthy donors and COVID-19 patients in validation study.

**Figure legend**

**Figure S1**. **Clinical factors at admission for early prediction of COVID-19 severity in discovery cohort.** (**a**) Patient recruitment flowchart for this cohort. (**b**) Correlations of age, smoking index, CRP, and ALT between the three subject groups. *P* values for trend by *Pearson’s* correlation analysis. Error bars represent mean ± SEM. (**c**) The AUC (95% CI) for age, smoking index, CRP, and ALT evaluated by ROC analysis. (**d**) Kaplan-Meier curves for age, smoking index, CRP, and ALT by Log-rank test. Time represents the number of days from admission to time of onset for a severe COVID-19 related event. In each case, optimal cut-off values were used to define high and low groups.

**Figure S2. Characterization of immunocaptured EVs for discovery set analysis.** (**a**) Scanning electron microscope (SEM) images of the immunocaptured EVs by beads. (**b**) Western blotting analysis of CD9 expression levels of 190 ng or 380 ng serum-derived EV protein isolated by immunoprecipitation (IP) or ultracentrifugation (UC). M: molecular marker. (**c, d**) % of ApoE (**c**) or ApoA-1 (**d**) in extracted EV protein fractions compared with serum samples (*n*=3).

**Figure S3**. **Characterization of isolated EVs by spin column with porous glass filter for validation set analysis.** (**a, b**) % of LDL/VLDL (**a**) or HDL (**b**) in extracted EV protein fractions compared with serum samples (*n*=5). (**c**) Western blotting analysis of COPB2 expression levels in #1. LDL/VLDL, #2. HDL, or #3. lipoprotein-depleted serum EV fraction by the column. LDL/VLDL and HDL were purified from an aliquot of 400 μL serum sample (#1 and #2). EVs were isolated from the lipoprotein-depleted serum (#3). ApoB and ApoA-1 are the main protein component of LDL/VLDL and HDL, respectively.
