# Supplementary material for: Early prediction of COVID‐19 severity using extracellular vesicle COPB2
Source: J Extracell Vesicles. 2021 Jun 2;10(8):e12092. doi: 10.1002/jev2.12092 (PMC8172627; doi:10.1002/jev2.12092)
Supplement: Supplementary file 2 — Supporting information. [file JEV2-10-e12092-s001.pdf]

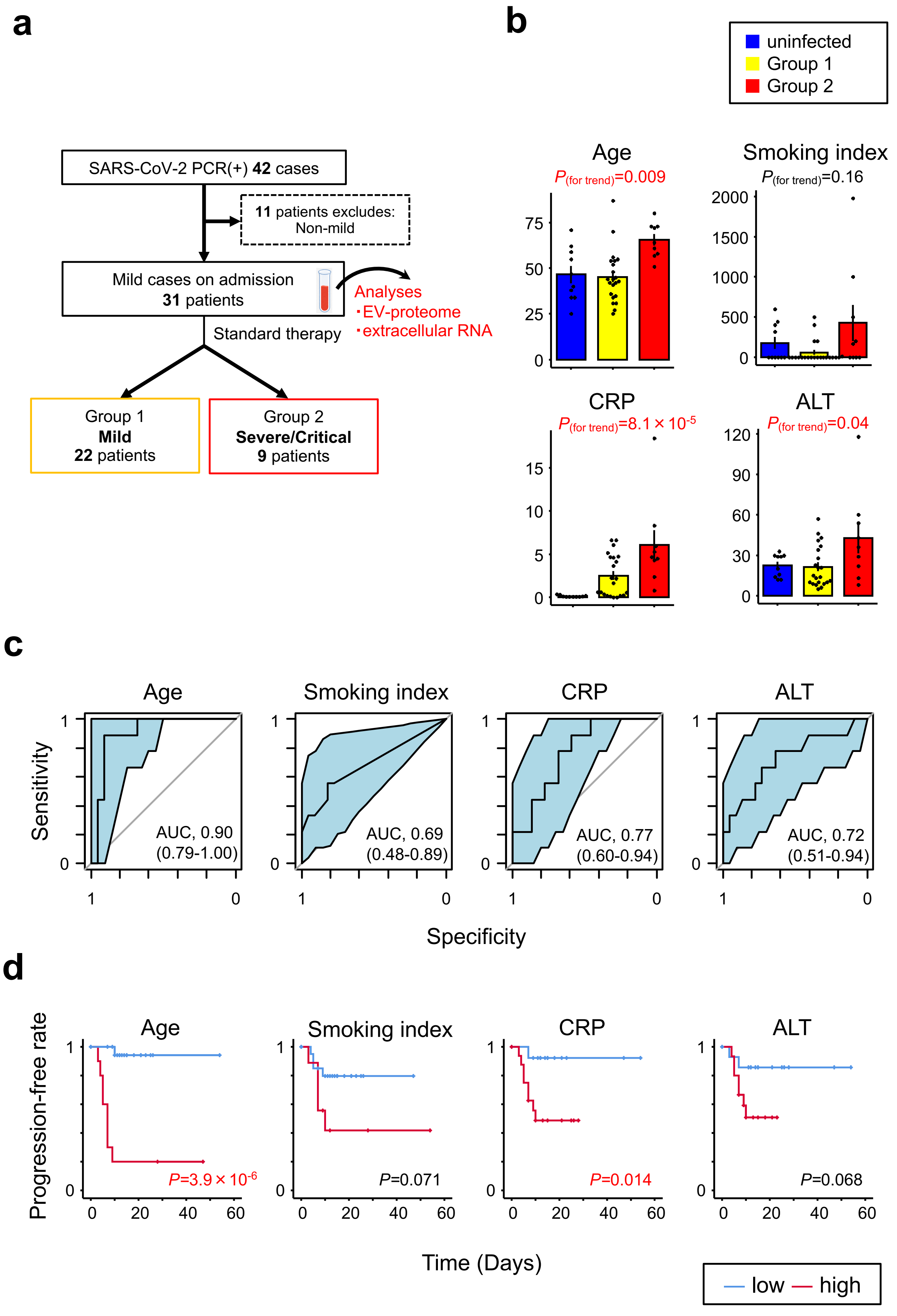

a
SARS-CoV-2 PCR(+) 42 cases
11 patients excludes: Non-mild
Mild cases on admission 31 patients
Analyses
・EV-proteome
・extracellular RNA
Standard therapy
Group 1 Mild 22 patients
Group 2 Severe/Critical 9 patients
b
uninfected
Group 1
Group 2
Age
P(for trend)=0.009
Smoking index
P(for trend)=0.16
CRP
P(for trend)=8.1×10-5
ALT
P(for trend)=0.04
c
Age
AUC, 0.90 (0.79-1.00)
Smoking index
AUC, 0.69 (0.48-0.89)
CRP
AUC, 0.77 (0.60-0.94)
ALT
AUC, 0.72 (0.51-0.94)
Sensitivity
Specificity
d
Progression-free rate
Age
P=3.9×10-6
Smoking index
P=0.071
CRP
P=0.014
ALT
P=0.068
Time (Days)
low
high

Figure S1

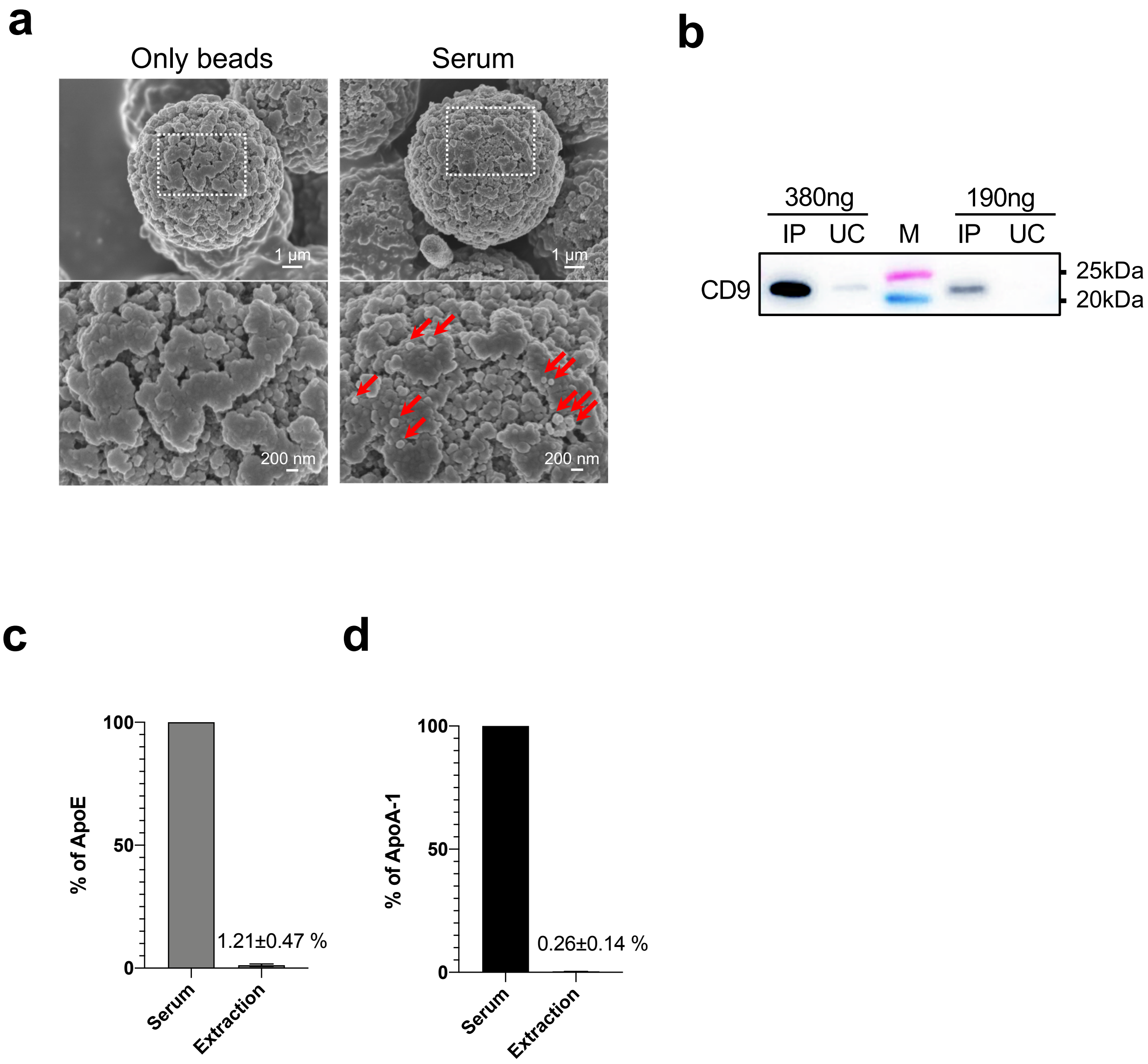
a
Only beads
Serum
1 µm
1 µm
200 nm
200 nm
b
380ng
190ng
IP
UC
M
IP
UC
CD9
25kDa
20kDa
c
100
50
0
% of ApoE
1.21±0.47 %
Serum
Extraction
d
100
50
0
% of ApoA-1
0.26±0.14 %
Serum
Extraction

Figure S2

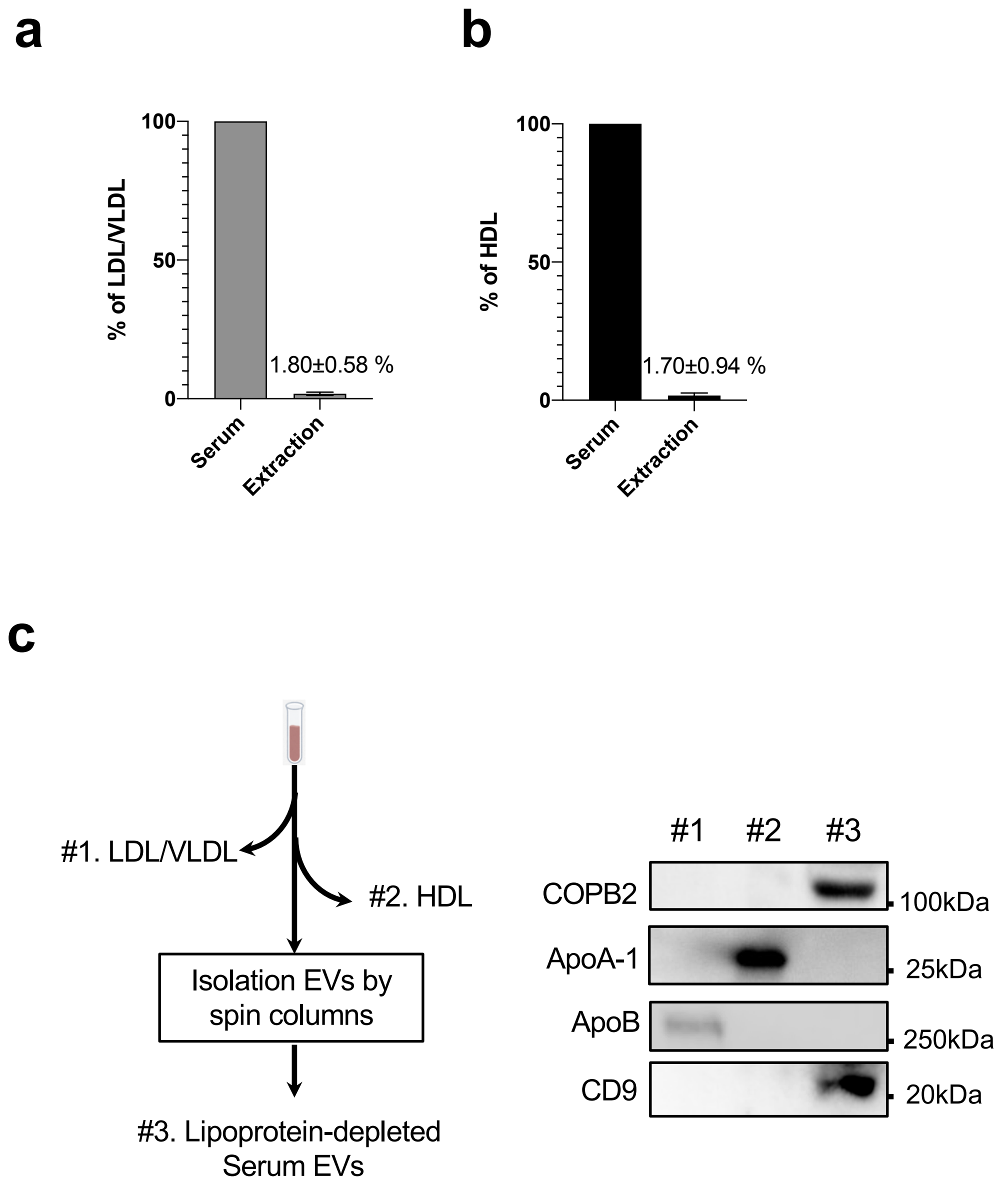
a
100
50
0
% of LDL/VLDL
1.80±0.58 %
Serum
Extraction
b
100
50
0
% of HDL
1.70±0.94 %
Serum
Extraction
c
#1. LDL/VLDL
#2. HDL
Isolation EVs by spin columns
#3. Lipoprotein-depleted Serum EVs
#1
#2
#3
COPB2
100kDa
ApoA-1
25kDa
ApoB
250kDa
CD9
20kDa

Figure S3

Table S1. Baseline characteristics of healthy donors and COVID-19 patients in discovery study.

	Patients	WHO category	Age	Sex	Onset of severe events (day)†	The period of hospital stay	Outcome
Healthy donor	HD-1	uninfected	38	M	-	-	-
	HD-2	uninfected	34	M	-	-	-
	HD-3	uninfected	25	F	-	-	-
	HD-4	uninfected	40	M	-	-	-
	HD-5	uninfected	71	M	-	-	-
	HD-6	uninfected	55	M	-	-	-
	HD-7	uninfected	62	M	-	-	-
	HD-8	uninfected	47	M	-	-	-
	HD-9	uninfected	59	M	-	-	-
	HD-10	uninfected	34	F	-	-	-
Group 1 COVID-19	1	mild→mild	34	M	-	15	discharged
	2	mild→mild	31	M	-	14	discharged
	3	mild→mild	48	M	-	10	discharged
	4	mild→mild	43	M	-	13	discharged
	5	mild→mild	52	M	-	21	discharged
	6	mild→mild	54	M	-	26	discharged
	7	mild→mild	87	M	-	28	discharged
	8	mild→mild	55	F	-	25	discharged
	9	mild→mild	25	F	-	11	discharged
	10	mild→mild	27	F	-	23	discharged
	11	mild→mild	42	F	-	15	discharged
	12	mild→mild	45	M	-	12	discharged
	13	mild→mild	70	M	-	47	discharged
	14	mild→mild	44	M	-	54	discharged
	15	mild→mild	44	M	-	9	discharged
	16	mild→mild	56	M	-	15	discharged
	17	mild→mild	31	F	-	12	discharged
	18	mild→mild	54	M	-	18	discharged
	19	mild→mild	41	F	-	21	discharged
	20	mild→mild	35	M	-	0	to medical facility
	21	mild→mild	36	F	-	0	to medical facility
	22	mild→mild	42	M	-	7	discharged
Group 2 COVID-19	23	mild→ critical	61	M	5	93	discharged
	24	mild→ critical	80	M	4	23	death (pulmonary embolism)
	25	mild→ critical	64	M	3	138	still hospitalized
	26	mild→ critical	72	M	9	44	discharged
	27	mild→ critical	73	M	7	29	discharged
	28	mild→ critical	73	M	7	125	still hospitalized
	29	mild→ critical	51	M	10	43	death (ARDS)
	30	mild→ severe	58	M	5	31	discharged
	31	mild→ severe	57	M	7	16	discharged

M, male; F, female; † Day after sampling point on admission.

Table S2. Differences in clinical characteristics among healthy donors and COVID-19 patients in discovery study.

	**uninfected** (n=10)	**Group 1 COVID-19** (n=22)	**Group 2 COVID-19** (n=9)	***P* value**	
				(uninfected vs. infected)	(Group 1 vs. Group 2)
Age (year)	46.5 ± 14.8	45.3 ± 14.3	65.4 ± 9.5	0.42	0.001
Sex					
Men	8 (80%)	15 (68.2%)	9 (100%)	1.00	0.08
Women	2 (20%)	7 (31.8%)	0 (0%)		
BMI (kg/m^2)	22.8 ± 4.4	23.0 ± 3.0 (n=20)	22.4 ± 3.2	0.95	0.63
Smoking index	176 ± 237	60 ± 140	427 ± 670	0.94	0.02
WBC count (10^3/μL)	6.7 ± 2.9	4.6 ± 1.5	5.2 ± 3.2	0.03	0.46
CRP (mg/dL)	0.12 ± 0.12	2.50 ± 2.41	6.07 ± 5.11	0.01	0.01
BUN (mg/dL)	13.7 ± 4.5	17.0 ± 16.6	22.6 ± 14.6	0.35	0.39
Cr (mg/dL)	0.84 ± 0.13	1.72 ± 3.22	1.88 ± 2.4	0.33	0.90
ALT (IU/L)	22.5 ± 8.5	21.4 ± 15.2	42.6 ± 33.2	0.51	0.02
CK (IU/L)	NA	69.5 ± 44.1	71.8 ± 50.8	NA	0.90
D-dimer (μg/mL)	NA	1.8 ± 2.0 (n=12)	1.1 ± 0.4	NA	0.28
Fbg (mg/dL)	NA	389.0 ± 84.2 (n=7)	480.4 ± 172.2	NA	0.22
Hypertension	2 (20.0%)	6 (27.3%)	3 (33.3%)	0.70	1.00
Diabetes mellitus	0 (0%)	5 (22.7%)	3 (33.3%)	0.17	0.66
Dyslipidemia	0 (0%)	4 (18.2%)	3 (33.3%)	0.16	0.38
Colonary heart disease	0 (0%)	2 (9.1%)	0 (0%)	1.00	1.00

Continuous variables were expressed as mean ± SD and tested by unpaired Student's *t*-test.
Categorical variables were expressed as n (%) and tested by Fisher's exact test.

Table S3. EV proteins for discrimination between mild and severe COVID-19 patients in discovery study.

GeneName	Cross-validation score	Sensitivity	Specificity	Accuracy	AUC
COPB2	1.00	1.00	1.00	1.00	1.00
KRAS	0.90	1.00	0.82	0.87	0.93
PRKCB	0.90	0.89	0.95	0.94	0.93
RHOC	0.90	0.78	1.00	0.94	0.96
CD147	0.87	0.67	0.91	0.84	0.73
CAPN2	0.87	0.89	0.77	0.81	0.84
ECM1	0.87	0.67	0.95	0.87	0.82
FGG	0.87	0.67	1.00	0.90	0.87
MFAP4	0.87	0.56	1.00	0.87	0.75
ADI1	0.84	0.67	0.95	0.87	0.88
AK1	0.84	0.89	0.91	0.90	0.95
MGAT1	0.84	1.00	0.77	0.84	0.91
CLDN3	0.84	0.89	0.82	0.84	0.86
CRP	0.84	0.78	0.77	0.77	0.82
UQCRC2	0.84	0.78	0.82	0.81	0.77
FGA	0.84	0.67	0.95	0.87	0.88
FGB	0.84	0.67	1.00	0.90	0.84
FGL1	0.84	0.78	0.82	0.81	0.85
GPX1	0.84	0.67	0.91	0.84	0.81
GSK3B	0.84	0.44	1.00	0.84	0.73
LBP	0.84	0.78	0.86	0.84	0.82
PDGFC	0.84	0.89	0.77	0.81	0.86
RAB13	0.84	0.78	0.86	0.84	0.85
RAP1B	0.84	0.67	1.00	0.90	0.91
SLC6A4	0.84	0.89	0.82	0.84	0.90
UBA7	0.84	0.78	0.86	0.84	0.83
ORM1	0.81	0.89	0.59	0.68	0.80
RNPEP	0.81	0.56	0.95	0.84	0.68
ANGPT1	0.81	0.78	0.86	0.84	0.88
APOB	0.81	0.78	0.86	0.84	0.79
B4GALT1	0.81	0.67	0.95	0.87	0.79
BHMT	0.81	0.44	1.00	0.84	0.74
CPN1	0.81	0.89	0.68	0.74	0.84
GNAZ	0.81	1.00	0.95	0.97	0.99
ICAM2	0.81	1.00	0.59	0.71	0.83
SELL	0.81	0.67	0.91	0.84	0.74
MAN1A1	0.81	0.78	0.82	0.81	0.85
SERPINA5	0.81	0.89	0.82	0.84	0.81
PACSIN2	0.81	0.89	0.86	0.87	0.90
NCF1B	0.81	0.89	0.55	0.65	0.73
TMEM59	0.81	0.44	0.95	0.81	0.59
YWHAB	0.77	0.67	0.95	0.87	0.85
ABAT	0.77	0.33	1.00	0.81	0.52
ADH1B	0.77	0.67	0.95	0.87	0.79
ASL	0.77	0.67	0.91	0.84	0.80
ASS1	0.77	0.78	0.86	0.84	0.79
CDH2	0.77	0.56	0.95	0.84	0.69
CAB39	0.77	0.89	0.91	0.90	0.94
CPS1	0.77	0.67	0.82	0.77	0.74
CD226	0.77	0.67	1.00	0.90	0.87
COL6A3	0.77	0.67	0.86	0.81	0.82
CUL4A	0.77	0.78	0.64	0.68	0.75
DSC1	0.77	0.44	0.95	0.81	0.58
ENTPD5	0.77	1.00	0.64	0.74	0.86
EIF4A1	0.77	0.67	0.86	0.81	0.80
FN1	0.77	0.89	0.68	0.74	0.81
PGC	0.77	0.89	0.68	0.74	0.78
RHEB	0.77	1.00	0.59	0.71	0.84
GNAI2	0.77	0.89	0.59	0.68	0.79
GNB1	0.77	0.78	0.77	0.77	0.83
GNA13	0.77	0.67	0.95	0.87	0.86
ITGA2B	0.77	0.67	0.91	0.84	0.86
ITGB1	0.77	1.00	0.59	0.71	0.84
ILK	0.77	0.89	0.77	0.81	0.84
F11R	0.77	1.00	0.50	0.65	0.82
LTA4H	0.77	0.56	0.91	0.81	0.67
LIMS1	0.77	0.89	0.77	0.81	0.83
NAV2	0.77	0.56	0.86	0.77	0.73
FAM129B	0.77	0.78	0.86	0.84	0.84
NNMT	0.77	0.67	0.91	0.84	0.69
NID1	0.77	0.89	0.55	0.65	0.76
PPIA	0.77	0.78	0.77	0.77	0.86
PLA1A	0.77	0.67	0.91	0.84	0.80
PPBP	0.77	0.67	0.77	0.74	0.69
PECAM1	0.77	1.00	0.55	0.68	0.82
GP1BB	0.77	0.67	0.86	0.81	0.81
PCSK9	0.77	1.00	0.82	0.87	0.91
MENT	0.77	0.44	0.95	0.81	0.71
SERPINA10	0.77	0.67	0.82	0.77	0.74
F2RL3	0.77	0.89	0.86	0.87	0.91
LOX	0.77	0.67	0.91	0.84	0.81
SFTPB	0.77	0.67	0.77	0.74	0.78
RAB5B	0.77	0.78	0.86	0.84	0.84
RALB	0.77	0.89	0.68	0.74	0.82
REEP6	0.77	0.78	0.68	0.71	0.71
RETN	0.77	0.67	0.73	0.71	0.69
AGXT	0.77	0.22	1.00	0.77	0.48
CCT2	0.77	0.89	0.64	0.71	0.81
THBD	0.77	0.56	0.91	0.81	0.74
ISG15	0.77	0.33	1.00	0.81	0.67
ZYX	0.77	1.00	0.55	0.68	0.82

Table S4. ExRNAs for discrimination between mild and severe COVID-19 patients in discovery study.

Transcript	Cross-validation score	Sensitivity	Specificity	Accuracy	AUC
miR-122-5p	0.84	0.56	1.00	0.87	0.81
SNORD33	0.84	1.00	0.73	0.81	0.89
AL732437.2	0.84	0.89	0.68	0.74	0.80
RNU2-29P	0.81	0.44	1.00	0.84	0.70
CDKN2B-AS1	0.81	0.67	0.95	0.87	0.86
AL365184.1	0.81	0.78	0.86	0.84	0.87
AL365184.1	0.81	0.78	0.91	0.87	0.90
AL365184.1	0.81	0.67	0.91	0.84	0.87
AL365184.1	0.81	0.78	0.91	0.87	0.90
AL365184.1	0.81	1.00	0.73	0.81	0.92
let-7c-5p	0.77	0.44	0.95	0.81	0.75
miR-21-5p	0.77	0.56	0.91	0.81	0.71
miR-140-3p	0.77	0.89	0.77	0.81	0.89
C5orf66-AS2	0.77	0.78	0.91	0.87	0.89

Table S5. Univariate Cox regrssion analysis of the selected factors and biomarkers for predicting COVID-19 severity in discovery study.

	Cut-off	HR	(95% CI)	P value
Age high	56.5	28.1	(3.4 - 231.9)	0.0019
Smoking index high	87.5	3.1	(0.8 - 11.6)	0.092
CRP high	2.3	8.4	(1 - 67.5)	0.045
ALT high	21.5	3.8	(0.8 - 18.5)	0.095
COPB2 low	1.6×10^{8}	NA	-	-
KRAS low	4.8×10^{7}	189.8	(0.4 - 9.0 $\times 10^{4}$)	0.095
PRKCB low	6.3×10^{7}	32.1	(3.9 - 261.9)	0.0012
RHOC low	1.4×10^{7}	23.6	(4.7 - 118)	0.00012
CD147 high	6.1×10^{8}	10.7	(2.5 - 45.1)	0.0013
CAPN2 high	7.2×10^{5}	15.5	(1.9 - 125.9)	0.010
ECM1 high	2.3×10^{8}	11.6	(2.8 - 48.4)	0.00079
FGG high	1.4×10^{10}	21.4	(4.2 - 110.4)	0.00025
MFAP4 high	4.6×10^{8}	12.7	(3.3 - 48.6)	0.00022
miR-122-5p high	1.0×10^{5}	10.5	(2.7 - 40.4)	0.00063
SNORD33 high	406.6	104.1	(0.4 - 2.7 $\times 10^{4}$)	0.10
AL732437.2 high	8.8	9.9	(1.2 - 79.9)	0.031
RNU2-29P high	124.1	10.4	(2.6 - 40.8)	0.00081
CDKN2B-AS1 high	6.2	14.4	(3.4 - 61.3)	0.00031
AL365184.1 high	2.9	14.2	(1.8 - 114.4)	0.013

HR, hazard ratio; CI, confidence interval; NA, not applicable

Table S6. Baseline characteristics of healthy donors and COVID-19 patients in validation study.

	Patients	WHO category	Age	Sex	Outcome
Healthy donor	JH001	uninfected	64	M	-
	JH002	uninfected	65	M	-
	JH003	uninfected	56	M	-
	JH004	uninfected	68	M	-
	JH005	uninfected	61	M	-
	JH006	uninfected	65	F	-
	JH007	uninfected	54	M	-
	JH008	uninfected	44	M	-
	JH009	uninfected	71	M	-
	JH010	uninfected	65	F	-
	JH011	uninfected	41	M	-
	JH012	uninfected	65	M	-
	JH013	uninfected	25	F	-
	JH014	uninfected	26	F	-
	JH015	uninfected	26	F	-
	JH016	uninfected	36	F	-
	JH017	uninfected	33	F	-
	JH018	uninfected	43	F	-
	JH019	uninfected	34	F	-
	JH020	uninfected	22	F	-
	JH021	uninfected	28	F	-
	JH022	uninfected	23	F	-
	JH023	uninfected	28	F	-
	JH024	uninfected	47	M	-
	JH025	uninfected	43	F	-
	JH026	uninfected	37	M	-
	JH027	uninfected	22	F	-
	JH028	uninfected	47	M	-
	JH029	uninfected	27	F	-
	JH030	uninfected	65	F	-
	JH031	uninfected	29	F	-
	JH032	uninfected	45	F	-
	JH033	uninfected	45	F	-
	JH034	uninfected	25	F	-
	JH035	uninfected	34	F	-
	JH036	uninfected	24	M	-
	JH037	uninfected	63	M	-
	JH038	uninfected	54	F	-
	JH039	uninfected	39	F	-

M, male; F, female.

	Patients	WHO category	Age	Sex	Outcome
Group 1 COVID-19	JC1	mild→mild	35	F	discharged
	JC2	mild→mild	73	M	discharged
	JC3	mild→mild	39	M	discharged
	JC4	mild→mild	53	F	discharged
	JC5	mild→mild	40	M	discharged
	JC6	mild→mild	29	M	discharged
	JC7	mild→mild	32	M	discharged
	JC8	mild→mild	21	F	discharged
	JC9	mild→mild	25	F	discharged
	JC10	mild→mild	52	M	discharged
	JC11	mild→mild	49	M	discharged
	JC12	mild→mild	46	M	discharged
	JC13	mild→mild	57	M	discharged
	JC14	mild→mild	59	M	discharged
	JC15	mild→mild	31	M	discharged
	JC16	mild→mild	22	M	discharged
	JC17	mild→mild	77	M	discharged
	JC18	mild→mild	64	M	discharged
	JC19	mild→mild	29	M	discharged
	JC20	mild→mild	33	M	discharged
	JC21	mild→mild	32	F	discharged
	JC22	mild→mild	65	F	discharged
	JC23	mild→mild	54	F	discharged
	JC24	mild→mild	34	M	discharged
	JC25	mild→mild	44	M	discharged
	JC26	mild→mild	24	F	discharged
	JC27	mild→mild	48	M	discharged
	JC28	mild→mild	68	M	discharged
	JC29	mild→mild	34	F	discharged
	JC30	mild→mild	43	M	discharged
	JC31	mild→mild	77	M	discharged
	JC32	mild→mild	47	M	discharged
	JC33	mild→mild	82	F	discharged
	JC34	mild→mild	30	F	discharged
	JC35	mild→mild	34	M	discharged
Group 2 COVID-19	JC45	mild→ severe	46	M	discharged
	JC47	mild→ critical	72	M	discharged
	JC48	mild→ severe	57	F	discharged
	JC50	mild→ severe	58	M	discharged
	JC51	mild→ severe	72	M	discharged
